# Structure–activity relationship of Cu-based catalysts for the highly efficient CO_2_ electrochemical reduction reaction

**DOI:** 10.3389/fchem.2023.1141453

**Published:** 2023-02-09

**Authors:** Runzhi An, Xuanqi Chen, Qi Fang, Yuxiao Meng, Xi Li, Yongyong Cao

**Affiliations:** ^1^ College of Biological, Chemical Science and Engineering, Jiaxing University, Jiaxing, Zhejiang, China; ^2^ College of Chemical Engineering, State Key Laboratory Breeding Base of Green-Chemical Synthesis Technology, Zhejiang University of Technology, Hangzhou, China

**Keywords:** electrochemical CO_2_ reduction reaction (CO_2_RR), Cu-based catalysts, density functional theory (DFT), size effect, compositional effect

## Abstract

Electrocatalytic carbon dioxide reduction (CO_2_RR) is a relatively feasible method to reduce the atmospheric concentration of CO_2_. Although a series of metal-based catalysts have gained interest for CO_2_RR, understanding the structure–activity relationship for Cu-based catalysts remains a great challenge. Herein, three Cu-based catalysts with different sizes and compositions (Cu@CNTs, Cu_4_@CNTs, and CuNi_3_@CNTs) were designed to explore this relationship by density functional theory (DFT). The calculation results show a higher degree of CO_2_ molecule activation on CuNi_3_@CNTs compared to that on Cu@CNTs and Cu_4_@CNTs. The methane (CH_4_) molecule is produced on both Cu@CNTs and CuNi_3_@CNTs, while carbon monoxide (CO) is synthesized on Cu_4_@CNTs. The Cu@CNTs showed higher activity for CH_4_ production with a low overpotential value of 0.36 V compared to CuNi_3_@CNTs (0.60 V), with *CHO formation considered the potential-determining step (PDS). The overpotential value was only 0.02 V for *CO formation on the Cu_4_@CNTs, and *COOH formation was the PDS. The limiting potential difference analysis with the hydrogen evolution reaction (HER) indicated that the Cu@CNTs exhibited the highest selectivity of CH_4_ among the three catalysts. Therefore, the sizes and compositions of Cu-based catalysts greatly influence CO_2_RR activity and selectivity. This study provides an innovative insight into the theoretical explanation of the origin of the size and composition effects to inform the design of highly efficient electrocatalysts.

## 1 Introduction

With the rapid development of the global society, the increasing emissions of carbon dioxide (CO_2_) from the burning of fossil fuels have led to serious environmental and energy crises (Jiao, 2019; Cao et al., 2021; Wang et al., 2022). The electrocatalytic CO_2_ reduction reaction (CO_2_RR) provides a viable alternative to convert CO_2_ into value-added chemical products such as carbon monoxide (CO), methane (CH_4_), methanol (CH_3_OH), ethylene (CH_2_CH_2_), and ethanol (CH_3_CH_2_OH) (Xue et al., 2020; Zhang, 2022; Zhou et al., 2022). Therefore, this is a promising approach to alleviate the global demand for fossil fuels and greenhouse effects. In addition, the advantages of CO_2_RR include 1) the feasibility of controlling the process by adjusting the potential and related conditions; 2) electricity consumption from sustainable sources such as the Sun and wind; 3) electrochemical reaction systems that are compact, modular, and easily scaled-up (Gong et al., 2019; Jiao et al., 2019; Bai et al., 2020; Han et al., 2020; He et al., 2020). However, there remain several challenges to the practical application of CO_2_RR, such as slow reaction kinetics, poor efficiency, and low product selectivity due to the strong C=O double bond (806 kJ.mol^-1^) in CO_2_ molecules (Alvarez et al., 2017). Therefore, highly active electrocatalysts must be designed to improve the sluggish kinetics and energy conversion efficiency.

A recent study demonstrated the high activity and selectivity of transition metal catalysts in the conversion of CO_2_ to CO, CH_4_, or CH_3_OH. Among these, Cu-based catalysts have been widely applied to CO_2_RR due to their excellent conductivity and availability (Li et al., 2017). The catalyst size and composition structure are usually regulated to tune the catalytic activity and selectivity of metal nanoparticles. Varying particle size has size effects in CO_2_RR. Studies have reported on the CO_2_RR trends over spherical Cu nanoparticles ranging in size from 2 to 15 nm (Shen et al., 2021). A decrease in Cu nanoparticle size leads to a significant increase in low coordination sites, resulting in strong CO_2_ adsorption and hydrogenation. A series of different sizes of Ni-based catalysts (Ni@CNT, Ni_4_@CNT, and Ni (110)) were also designed to study their size effects in CO_2_RR. Density functional theory (DFT) calculations revealed that the Ni@CNT catalyst is much more conducive to the conversion of CO_2_ to CO, while CH_4_ molecules were produced on Ni@CNT and Ni (100) with increasing Ni size (Cao et al., 2020a). Therefore, the catalyst size may affect CO_2_ activation and final products.

The alloy effect of the metal catalyst is another key factor in catalytic reactions. The electronic structure of metal catalysts can be controlled by alloying with other metal atoms, which is an obvious characteristic of Cu-based catalysts (Weng et al., 2018; Meng et al., 2022). A Cu–Ni alloy catalyst embedded in the N-doped carbon framework (CuNi/NC) synthesized for CO_2_RR showed excellent activity and selectivity for electrocatalytic CO_2_RR to CO, with a high Faradaic efficiency of 99.7% and a potential of −0.6 V (Zhang, 2019; Zhong et al., 2020). Additionally, a non-noble metal CuSn alloy with a different Cu–Sn composition demonstrated a completely different distribution of products for CO_2_RR, including CO and formate (Li et al., 2020a). Tuning the surface composition of Cu-based catalysts *via* alloying with other metals could directly modify the absorbed state of the intermediate species on their active sites and determine the final product during the CO_2_RR. Therefore, the introduction of other metals is an effective strategy to adjust the electronic structure and improve CO_2_RR activity and selectivity.

This study designed different sizes and compositions of Cu-based catalysts (Cu@CNT, Cu_4_@CNT, and CuNi_3_@CNT) to explore the relationship between the structure of catalysts and activity for CO_2_RR based on DFT calculations. CO_2_ molecule adsorption and activation are essential for the following hydrogenation steps. Therefore, different CO_2_ adsorption configurations were optimized to explore the nature of CO_2_ activation on the three catalysts by analyzing their geometric and electronic properties. The mechanisms of CO_2_RR on different Cu-based catalysts were also assessed, and a plot was constructed to compare the selectivity of CO_2_RR among Cu@CNT, Cu_4_@CNT, and CuNi_3_@CNT.

## 2 Computational models and methods

All calculations were performed using the Vienna *Ab initio* Simulation Package (VASP) (Kresse and Hafner, 1993; Kresse and Furthmiiller, 1996), including the Perdew–Burke–Ernzerhof (PBE) functions in the generalized gradient approximation (GGA) to describe electron exchange and correction effect (Blochl, 1994; Perdew et al., 1996; Kresse and Joubert, 1999). The cutoff energy for the plane wave basis was set to 520.0 eV. The Brillouin zone was sampled at 4 × 4 × 1 *k* points for all calculation models using the Monkhorst–Pack grid. The convergence criteria for energy for geometry optimization were set to 1.0 × 10^−5^ eV. The vacuum thickness was 15.0 Å to prevent interlayer interactions. van der Waals correction was implemented using the Grimme’s DFT-D3 method (Krieg, 2010).

The adsorption energy ( $E_{ads}$ ) of CO_2_ is calculated using the following equation:

$$E_{ads}=E_{cat+mol}\;–\;E_{cat}\;–\;E_{mol},$$ (1)

where $E_{cat+mol}$ is the total energy of CO_2_ molecule or intermediate adsorption on Cu@CNTs, Cu_4_@CNTs, and CuNi_3_@CNTs; $E_{cat}$ is the optimized energy of three catalysts; and $E_{mol}$ is the energy of the adsorbed molecule.

The binding energy ( $E_{b}$ ) of a single Cu atom, Cu_4_, and CuNi_3_ cluster anchored on N-doped CNT is defined as follows:

$$E_{b}=E_{total}\;–E_{N-CNT}\;–E_{metal},$$ (2)

where $E_{total}$ is the total energy of Cu@CNTs, Cu_4_@CNTs, and CuNi_3_@CNTs; $E_{N-CNT}$ denotes the optimized energy of N-doped CNT support; and $E_{metal}$ is the energy of a single Cu atom or Cu_4_ or the Cu_3_Ni cluster.

The computational hydrogen electrode (CHE) mode was proposed by Nørskov et al (Nadolska et al., 2021), in which the changes in Gibbs free energies of CO_2_RR are calculated for every elementary step. The reference electrode is the reversible hydrogen electrode (RHE). Therefore, the chemical potential (μ) of the proton–electron pair is defined as follows:

$$\mu_{H^{+}}+\mu_{e^{-}}\;\to \;\mu_{H_{2}}\;\left(g\right),$$ (3)

The change in Gibbs free energy ( ∆G ) for each elementary step is determined by the following equation:

$$∆G=∆E+∆E_{ZPE}\;–\;T∆S+∆G_{U},$$ (4)

where $∆E_{DFT}$ is the change of electron energy calculated by the DFT calculation, $∆E_{ZPE}$ is the change of zero-point correction energy, ∆S is the change of the entropy gradient, and *T* is the temperature.

$$∆G_{U}=–neU,$$ (5)

where *e* is the fundamental charge transfer, *n* is the proton–electron logarithm transferred, and *U* is the electrode applied potential relative to the RHE.

The overpotential (*η*) of CO_2_RR is defined as follows:

$$\eta =U_{equilibrium}\;–\;U_{limiting},$$ (6)

where $U_{equilibrium}$ is the equilibrium potential of CO_2_RR. The limiting potential $U_{limiting}$ is calculated as *U*_limiting_ = –Δ*G/e*, where Δ*G*_limiting_ represents the free energy of the potential-limiting step.

**FIGURE 1:**
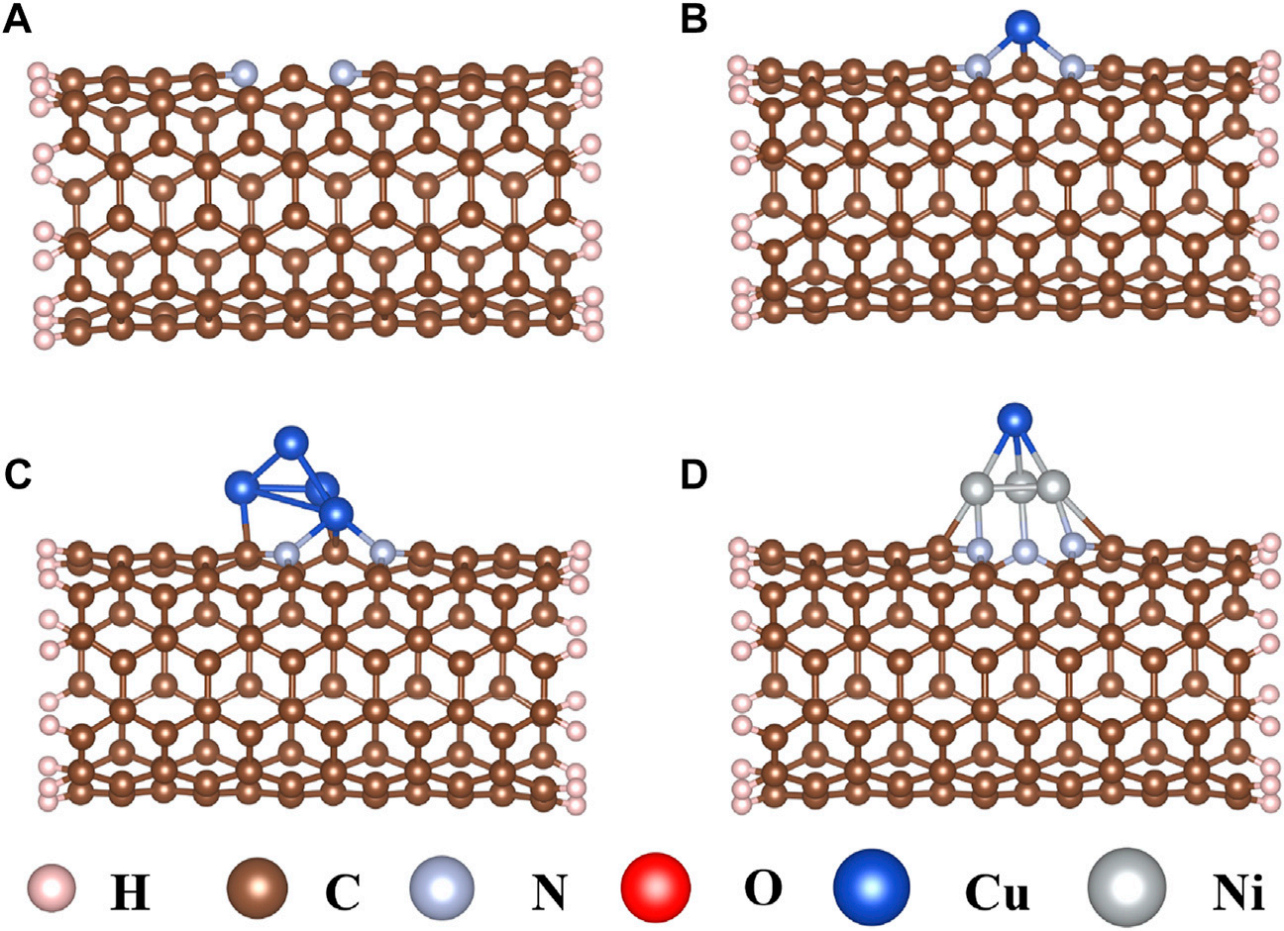
Optimized structures of **(A)** nitrogen-doped carbon nanotubes, **(B)** single Cu atom (Cu@CNTs), **(C)** Cu_4_ cluster (Cu_4_@CNTs), and **(D)** CuNi_3_ (CuNi_3_@CNTs) supported on the nitrogen-doped carbon nanotubes.

Carbon nanotubes (CNTs) are promising substitutes that have been widely applied in electrocatalysts for their high electrical conductivity, thermal stability, and abundant catalytic active sites (Grimme et al., 2010; Xiong et al., 2019; Bulmer et al., 2021; Cai et al., 2021). As an N-doped CNT (Figure 1A) may further increase interaction between CNT and the active center (Park et al., 2020; Nguyen and Shim, 2021), a single Cu atom, Cu_4_ cluster, and CuNi_3_ cluster were doped on the N-doped CNTs through the carbon vacancy defect to explore the effects on the CO_2_RR sizes and composition (Figures 1B–D). As shown in Figure 1B, the single Cu atom is connected to two N atoms and one C atom to form a pyramid-like structure with *E*_b_ values of −3.21 eV. The bond lengths of Cu–N and Cu–C are 2.11 Å and 1.80 Å, respectively. When the Cu_4_ cluster is doped on the N-doped carbon nanotubes, the three Cu atoms at the bottom interact with two N atoms and two C atoms (Figure 1C), resulting in a strong interaction with *E*_b_ values of −4.87 eV. The Cu–N, Cu–C, and Cu–Cu bond lengths are 2.05 Å, 2.03 Å, and 2.42 Å, respectively. In addition, CuNi_3_@CNTs are used to study the compositional effect of Cu-based catalysts for CO_2_RR by replacing three Cu atoms in Cu_4_@CNTs with three Ni atoms connected to the adjacent N atoms and C atoms on the bottom of the tetrahedron, respectively (Figure 1D). The binding energy of CuNi_3_@CNTs is −5.12 eV. The large binding energies indicate that the three different Cu-based catalysts all possess high thermodynamic stability, ensuring their long-term use.

## 3 Results and discussion

### 3.1 CO_2_ activation on Cu@CNTs, Cu_4_@CNTs, and CuNi_3_@CNTs

**FIGURE 2:**
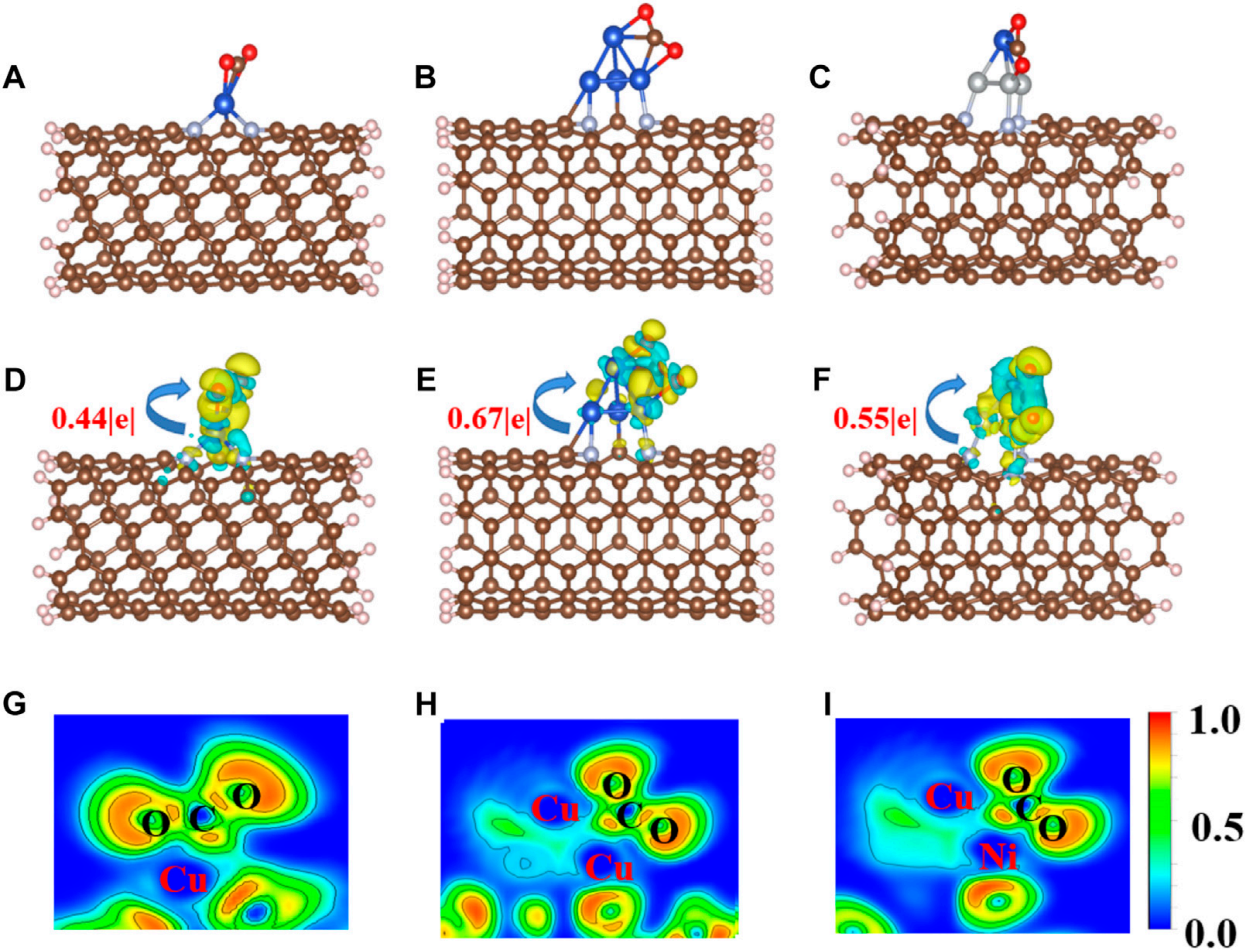
**(A–C)** Optimized adsorption configurations of CO_2_ on Cu@CNTs, Cu_4_@CNTs, and CuNi_3_@CNTs. **(D–F)** Calculated charge density difference (CDD) and **(G–I)** the electronic local function diagram (ELF) of CO_2_ adsorption. The charge depletion and accumulation are depicted in blue and yellow, respectively. The equivalent face value is 0.005 e/Å^–3^.

CO_2_ adsorption and activation are crucial steps for subsequent hydrogenation in CO_2_RR (Jiao et al., 2019). To investigate the behaviors of CO_2_ adsorption, we explored the geometric and electronic structures of the adsorbed CO_2_ molecules. As shown in Figure 2A, the CO_2_ molecule is adsorbed on the single Cu atom site with an *E*_ads_ value of −0.50 eV. The C=O bond length in CO_2_ is also slightly elongated from 1.18 to 1.25 Å. With increasing Cu active center size (from a single Cu atom to the Cu_4_ cluster), the *E*_ads_ value of CO_2_ decreases to −0.19 eV. The CO_2_ prefers to adsorb on the edge site of the Cu_4_ cluster (Figure 2B) compared to the top site, with an *E*_ads_ value of 0.15 eV. The adsorption site and configuration of CO_2_ on the CuNi_3_@CNTs (Figure 2C) are the same as that on the Cu_4_@CNTs, although the *E*_ads_ value increases to −0.82 eV. The C=O bond length also increases to 1.26 Å. The top adsorption site of CuNi_3_@CNTs has an adsorption energy of 0.35 eV (Supplementary Figure S1). Thus, the top adsorption site was used for CO_2_ molecule adsorption and activation on both the Cu_4_@CNTs and CuNi_3_@CNTs. The bond angles of O−C−O in the CO_2_ molecule changed from 180.00° to 146.74°, 140.77°, and 139.23° on Cu@CNTs, Cu_4_@CNTs, and CuNi_3_@CNTs, respectively (Table 1). Thus, Cu@CNTs, Cu_4_@CNTs, and CuNi_3_@CNTs all significantly activate CO_2_, and the size and composition effect greatly influence the degree of CO_2_ activation, which may further affect the activity and selectivity of CO_2_ hydrogenation.

**TABLE 1:** Geometric and electronic structure parameters of adsorbed *CO_2_ on the three Cu-based catalysts.

System	*E*_ads_ (eV)*^a^	Δ*Angle* (degree)*^b^	Δ*q* (|e|)*^c^	ICOHP (eV)*^d^
Cu@CNTs	−0.50	146.74	0.44	−4.47
Cu_4_@CNTs	−0.19	140.77	0.67	−4.43
CuNi_3_@CNTs	−0.82	139.23	0.55	−3.82

*a, adsorption energy of adsorbed CO_2_; *b, bond angle of adsorbed CO_2_; *c, number of electrons obtained by adsorbed *CO_2_; *d, values of the crystal integral Hamiltonian population (ICOHP) for the C and O atoms in the adsorbed *CO_2_ molecules.

To further investigate the activation mechanism of the CO_2_ molecules, the Bader charge, charge density difference (CDD), electronic local function diagram (ELF), projected density of state (PDOS), and integrated crystal orbital Hamilton population (ICOHP) with CO_2_ adsorption were investigated. As shown in Figures 2D–F, significant charge transfers occur between C=O double bonds and metal Cu and Ni atoms connected to CO_2_. In detail, the electron densities of the O atom, and between the C, O atoms and Cu, Ni atoms increase, while the electron density of the C=O double bond decreases significantly. In addition, the ELF map is used to further verify the bonding properties of C=O double bonds (Peterson et al., 2010; Reske et al., 2014; Wang et al., 2022). The results of the ELF calculation (Figures 2G–I) showed that the C=O double bond is weak, consistent with the results of the CDD analysis. The local electron densities of the Cu-C and Cu-O bonds range from 0.5 to 1.0 in the whole ELF map, which further verifies the metal coordination bond formation between Cu and Ni atoms and between the C and O atoms. The adsorbed *CO_2_ molecule gains 0.44|e|, 0.67|e|, and 0.55|e| from the Cu@CNTs, Cu_4_@CNTs, and CuNi_3_@CNTs, respectively, based on the Bader charge analysis, resulting in the bending of the bond angles of the adsorbed CO_2_ molecules.

**FIGURE 3:**
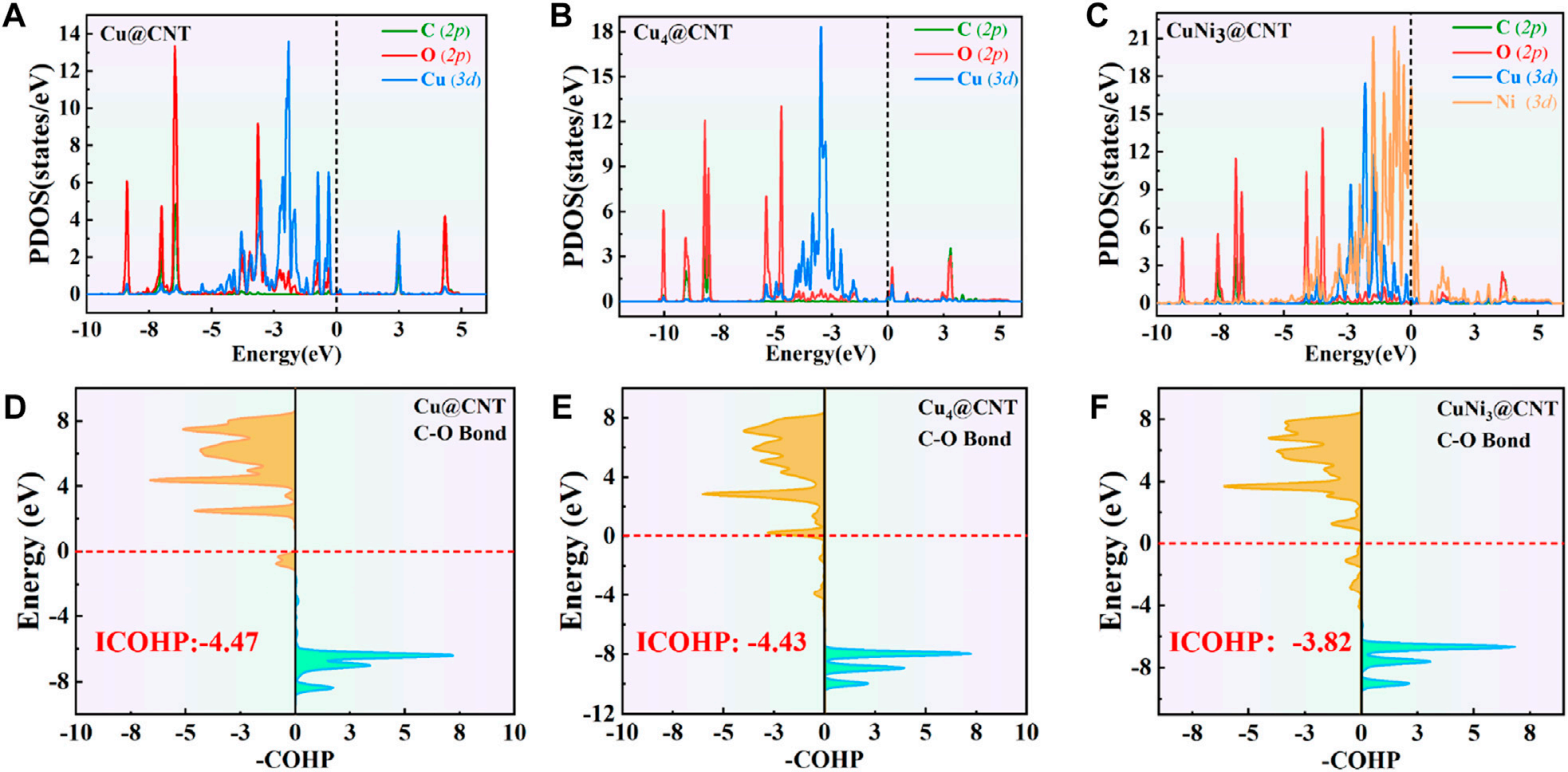
**(A–C)** Projected density of state (PDOS) plots. **(D–F)** Projected crystal orbital Hamilton population (pCOHP) of CO_2_ adsorption on Cu@CNTs, Cu_4_@CNTs, and CuNi_3_@CNTs. The dotted line represents the Fermi energy level.

The PDOS of CO_2_ adsorption are analyzed to further reveal interactions between Cu atoms and CO_2_ (Figures 3A–C). The Cu-3*d* and Ni-3*d* orbitals play key roles in CO_2_ adsorption and activation. The C-2*p*, O-2*p*, and Cu-3*d* orbitals have significant peak overlaps near the Fermi level, indicating strong interactions between C, O, and Cu atoms during CO_2_ adsorption on Cu@CNTs and Cu_4_@CNTs (Figures 3A,B). With the introduction of the Ni atom, the Cu-3*d* and Ni-3*d* orbitals show hybridization, illustrating the strong interaction between the Cu and Ni atoms. Meanwhile, the broadly overlapped regions by C-2*p*, O-2*p*, Cu-3*d*, and Ni-3*d* hybrids also appear near-Fermi level, with strong peak intensities (Figure 3C). Based on the CDD and PDOS results, the mechanism of CO_2_ activation can be summarized as follows: anti-bonding orbitals of CO_2_ accept 3*d*-electrons from the Cu-3*d* and Ni*-3d* orbitals; meanwhile, the π electrons from the CO_2_ bonding orbitals revert to empty Cu-3*d* and Ni*-3d* orbitals. The activation strength of the C=O double bond of the adsorbed CO_2_ molecule is quantitatively studied by calculating the ICOHP values, in which lower values indicate stronger bond strength (Li et al., 2021; Song et al., 2021). As seen in Figures 3D–F, the C=O double bond anti-bonding states move down relative to the Fermi level after CO_2_ adsorption. Therefore, the strength of the C=O double bond significantly decreases after CO_2_ adsorption. The ICOHP values of the C=O double bond are −4.47, −4.43, and −3.82 eV on Cu@CNTs, Cu_4_@CNTs, and CuNi_3_@CNTs, respectively. The weaker the C=O double bond, the more efficient the CO_2_ activation on the catalyst surface. This result demonstrates the higher degree of CO_2_ activation on the CuNi_3_@CNTs compared to that of the Cu@CNTs and Cu_4_@CNTs, consistent with the results of Bader charge, CDD, and adsorption energy calculations.

### 3.2 Mechanisms of CO_2_RR on Cu-based catalysts

CO_2_RR is a complex reaction involving multi-electron (two, six, or eight-electron) reaction pathways (Jiwanti and Einaga, 2019; Niu et al., 2021; Yang et al., 2022). The overall reaction equation can be expressed as follows:

$${CO}_{2}+*+{2H}^{+}+{2e}^{–}\;\to \;CO+H_{2}O+*$$ (7)

$${CO}_{2}+*+{6H}^{+}+{6e}^{–}\;\to \;{CH}_{3}OH+H_{2}O+*$$ (8)

$${CO}_{2}+*+{8H}^{+}+{8e}^{–}\;\to \;{CH}_{4}+{2H}_{2}O+*$$ (9)

**SCHEME 1:**
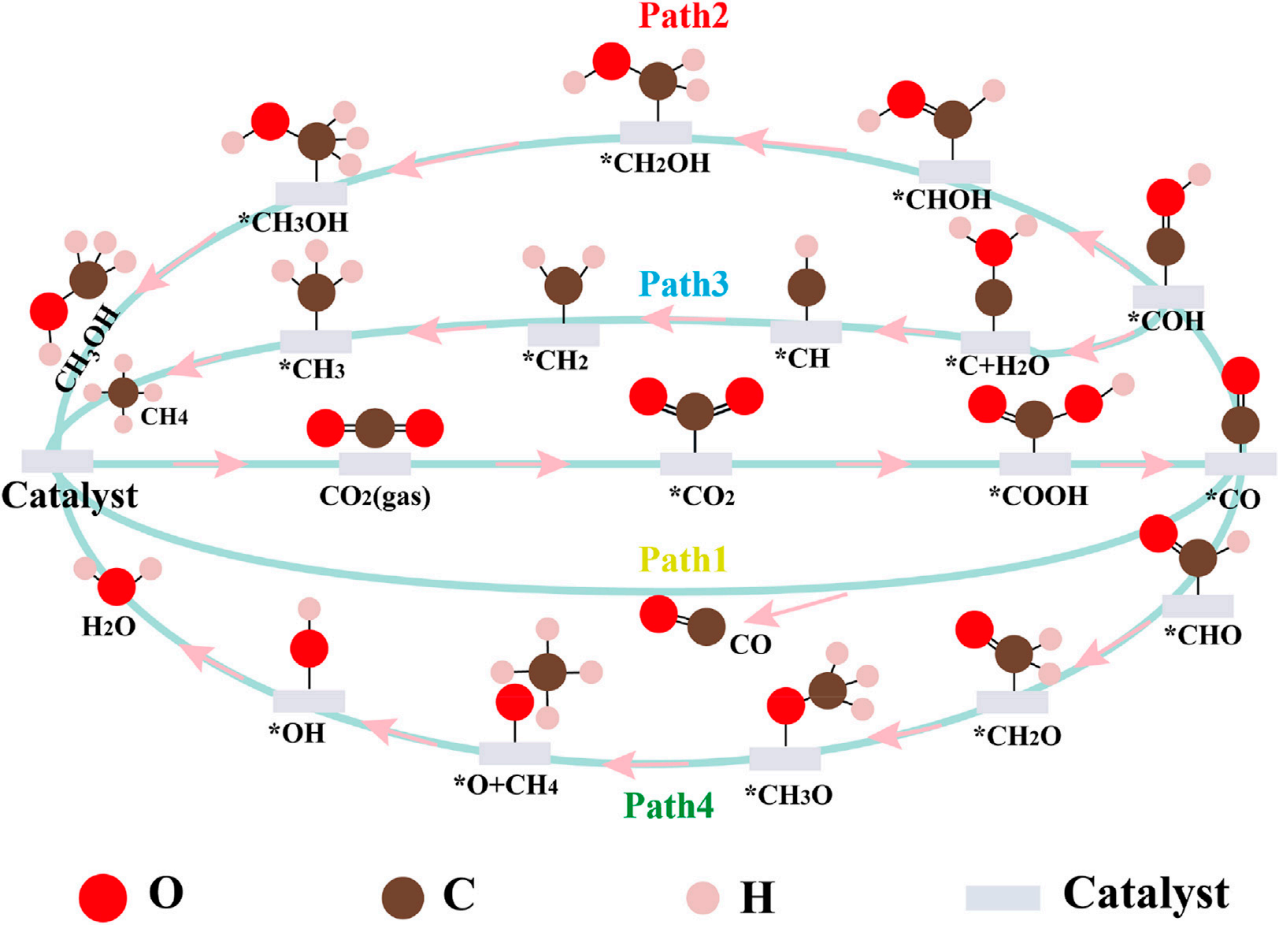
Proposed mechanisms of CO_2_RR to C_1_ products. *represents the catalytic surface.

Scheme 1 shows the different reaction paths for CO_2_RR to C1 products (CO, CH_4_, and CH_3_OH). According to the aforementioned description, the first step is CO_2_ adsorption. Then, an electron from the catalyst surface attacks the adsorbed *CO_2_ molecules to form the radical CO_2_^−^, resulting in the bending of the angle of the adsorbed *CO_2_. Subsequently, the radical CO_2_^−^ intermediate combines with the H atom to form the key *COOH intermediate. The *COOH intermediate then is further hydrogenated to form *CO and H_2_O. In Path 1, the adsorbed *CO diffuses to the gas phase. In the other paths, the adsorbed *CO species is further protonated to form *COH or *CHO *via* the formation of C-H or O-H bonds. Thus, CH_4_ and CH_3_OH products can be obtained by further hydrogenation of the key intermediates *COH or *CHO. In Path 2, CH_3_OH is synthesized starting from the *COH intermediate. The proton/electron pair (H^+^/e^−^) continuously attaches the C atom of *COH to form the *CHOH, *CH_2_OH, and *CH_3_OH intermediates. *CH_3_OH then desorbs to form the CH_3_OH product. In contrast, CH_4_ can be formed from *CHO or *COH *via* paths 3 and 4, respectively. In Path 3, the O atom of *COH is hydrogenated to form H_2_O and *C. In the next four hydrogenation steps, *CH, *CH_2_, *CH_3_, and CH_4_ are continuously generated. Moreover, the *CHO intermediate can eventually be protonated to produce CH_4_ by the *CH_2_O, *CH_3_O, O* + CH_4_, *OH, and *H_2_O species (Path 4). In addition, the C_2_ products also can be synthesized on the Cu-based catalysts. However, when two CO_2_ molecules are co-adsorbed on Cu@CNTs, Cu_4_@CNTs, and CuNi_3_@CNTs, only one CO_2_ molecule can be adsorbed on the single-atom Cu, Cu_4_ cluster, and CuNi_3_ cluster; the other CO_2_ molecule diffuses to the gas phase (Supplementary Figure S2). Therefore, this study explores only four paths for CO_2_RR to C1 products.

### 3.3 Reaction mechanism for CO_2_RR to CH_4_ products on Cu@CNTs

**FIGURE 4:**
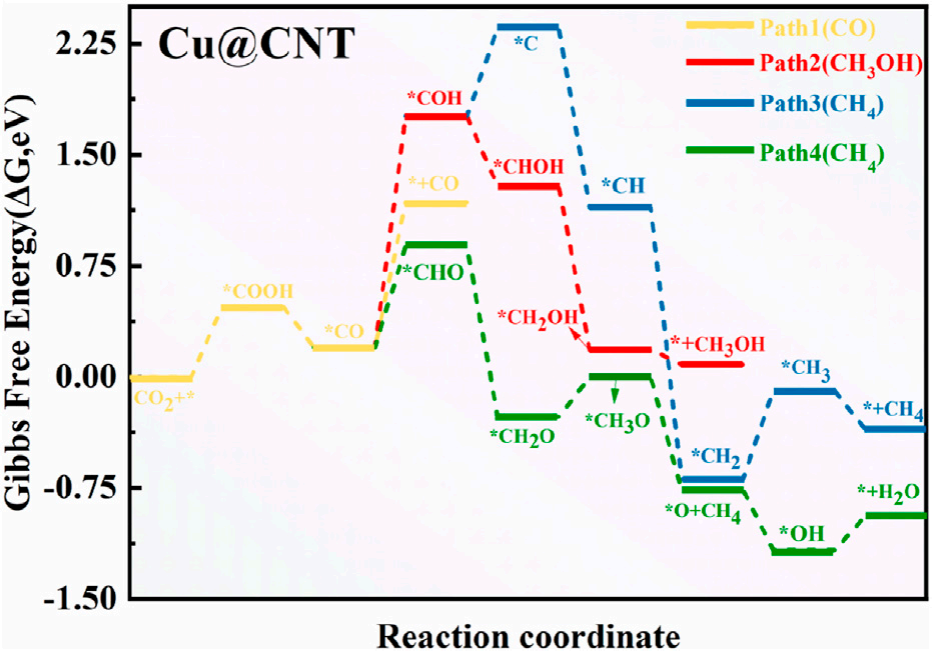
Free energy diagram of CO_2_RR on Cu@CNTs.

**FIGURE 5:**
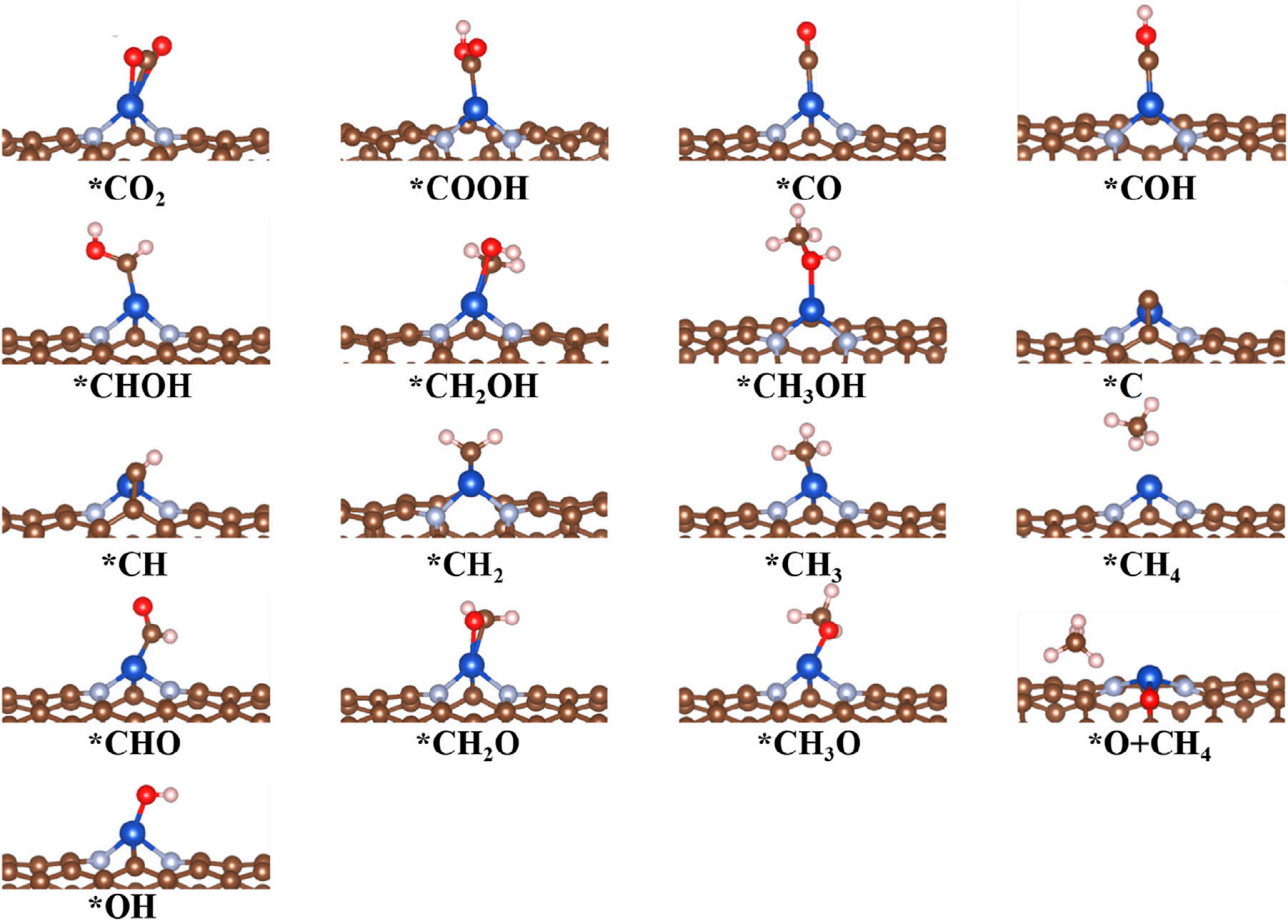
Optimized structures of intermediates for CO_2_RR on Cu@CNT surfaces.

To illustrate the catalytic activity and selectivity of CO_2_RR on Cu@CNTs, the free energy barrier diagram and corresponding intermediate configurations are shown in Figures 4, 5, respectively. Figure 4 shows that the absorbed CO_2_ molecule is hydrogenated to form stable *COOH with a change of the free energy value (Δ*G*) of 0.47 eV. In the second hydrogenation step, *COOH can react with the (H^+^/e^−^) pair to form *CO and H_2_O, with an exothermic energy release of –0.27 eV. The adsorbed *CO is further protonated to form *CHO (Δ*G =* 0.61 eV) and *COH (Δ*G =* 1.49 eV) intermediates, or may be directly desorbed with a Δ*G* value of 0.91 eV. Obviously, the formation of *CHO is more favorable than that of *CHO and *CO desorption. When the *COH species is formed, the C atom of *COH accepts three H atoms to form *CH_3_OH with downhill free energies of −0.47, −1.18, and −0.08 eV. *COH is also directly dissociated into *C species (Δ*G* = 0.60 eV), and *CH_4_ is formed by four hydrogenation steps of the *C intermediate. The formation of *CH and *CH_2_ is slightly thermodynamically favorable, with Δ*G* values of −1.22 and −1.83 eV, respectively. However, the formation of *CH_3_ has an endothermic energy of 0.78 eV. It is difficult to form the CH_4_ molecule in Path 3 due to the high hydrogenation barrier of the formation of *COH species for the whole reaction. In Path 4, *CHO intermediate accepts an (H^+^/e^−^) pair to form *CH_2_O with a Δ*G* value of −1.15 eV. Then, the hydrogenation of *CH_2_O to *CH_3_O is uphill by 0.26 eV. *CH_3_O is further hydrogenated to form *O and a CH_4_ molecule with a Δ*G* value of −0.75 eV. The leaving *O atom is continuously hydrogenated to form H_2_O. Thus, the conversion of CO_2_ to CH_4_ following Path 4 with an overpotential value of 0.37 V is easier than that of CO and CH_3_OH in all four paths, and the potential-determining step (PDS) is the *CHO formation on the Cu@CNTs. Wang et al. prepared three Cu-based catalysts for CO_2_RR (Weng et al., 2018). Among them, the single-atom Cu phthalocyanine catalyst exhibited the highest activity to produce CH_4_ with a Faradaic efficiency of 66% and a partial current density of 13 mA cm^−2^ at a potential of −1.06 V versus RHE, findings that are consistent with the calculation results of CO_2_RR to CH_4_ on the Cu-based single-atom catalysts in the present study.

### 3.4 Reaction mechanism for CO_2_RR to CO products on Cu_4_@CNTs

**FIGURE 6:**
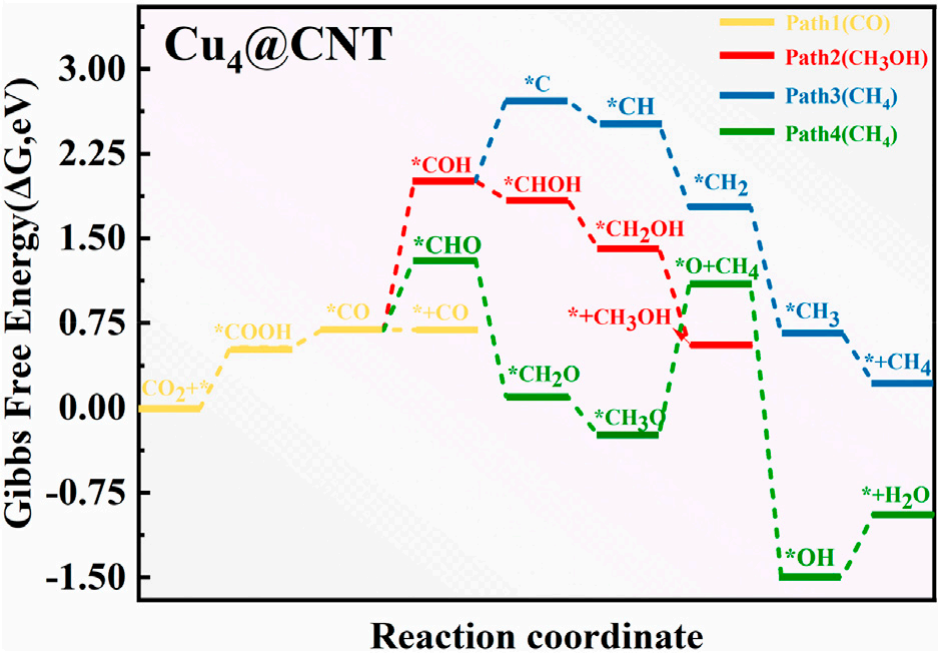
ree energy diagram of CO_2_RR on Cu_4_@CNTs.

**FIGURE 7:**
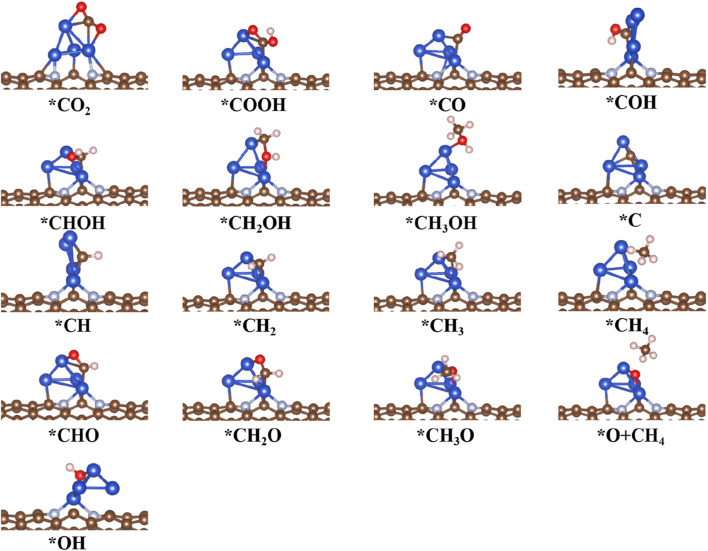
Optimized structure of intermediates for CO_2_RR on Cu_4_@CNT surfaces.

To explore the different size effects on CO_2_RR activity on Cu-based catalysts, the free energy profiles and optimized geometric structures for CO_2_RR on the Cu_4_@CNTs are shown in Figures 6, 7, respectively. The Cu_4_ clusters embedded in the CNTs catalyst provided multiple active sites for the activation of CO_2_ molecules compared to Cu@CNTs and further promoted hydrogenation. The adsorbed *CO_2_ accepts two (H^+^/e^−^) pairs to successively convert the key intermediates *COOH and *CO with an entirely endothermic process of 0.54 eV and 0.16 eV, respectively. CO desorption is a thermodynamically favorable process with a low energy of −0.01 eV compared to *CO hydrogenation to *COH (Δ*G =* 1.32 eV) and *CHO (Δ*G =* 0.62 eV). In the subsequent hydrogenation steps, the (H^+^/e^−^) pair consecutively attacks the C atom of the *CO intermediates to form *CHO, *CH_2_O, *CH_3_O, and *O+CH_4_ species, with PDS, the process of *O+ CH_4_ intermediate formation, with Δ*G* values of 1.32 eV in Path 4. After *COH formation, *COH hydrogen dissociates to form *C with an uphill energy of 0.72 eV. Then, the *C species is further hydrogenated to form *CH, *CH_2_, *CH_3_, and *CH_4_ (Path 3). The corresponding Δ*G* values are −0.21, −0.74, −1.11, and −0.44 eV, respectively. In Path 2, *COH is hydrogenated to form *CHOH, *CH_2_OH, and *CH_3_OH species, with Δ*G* values of −0.17, −0.43, and −0.86 eV. Comparison of the Δ*G* values of PDS in all four paths shows that the reactions are more likely to follow Path 1 and CO is produced on the Cu_4_@CNTs with an extremely low overpotential value of 0.02 V. More importantly, the desorption energy of CO is greatly reduced with increasing Cu size, which is conducive to the production of CO and avoids poisoning the active sites on the catalyst surface. In addition, the reported atom-pair catalyst with stable Cu_1_^0^–Cu_1_^x+^ pair structures also shows high selectivity and activity for CO_2_RR to CO (Li et al., 2020b). Therefore, our calculation results of CO_2_RR on cluster catalysts are consistent with experimental results reported previously. These results demonstrate that CO_2_RR reduction product changes from CH_4_ to CO when the size of Cu–based catalysts changes from a single atom to a Cu_4_ cluster. Therefore, designing multiple active sites to change the final reduction products may be a universal strategy for catalyst development.

### 3.5 Reaction mechanism for CO_2_RR to CH_4_ products on CuNi_3_@CNTs

**FIGURE 8:**
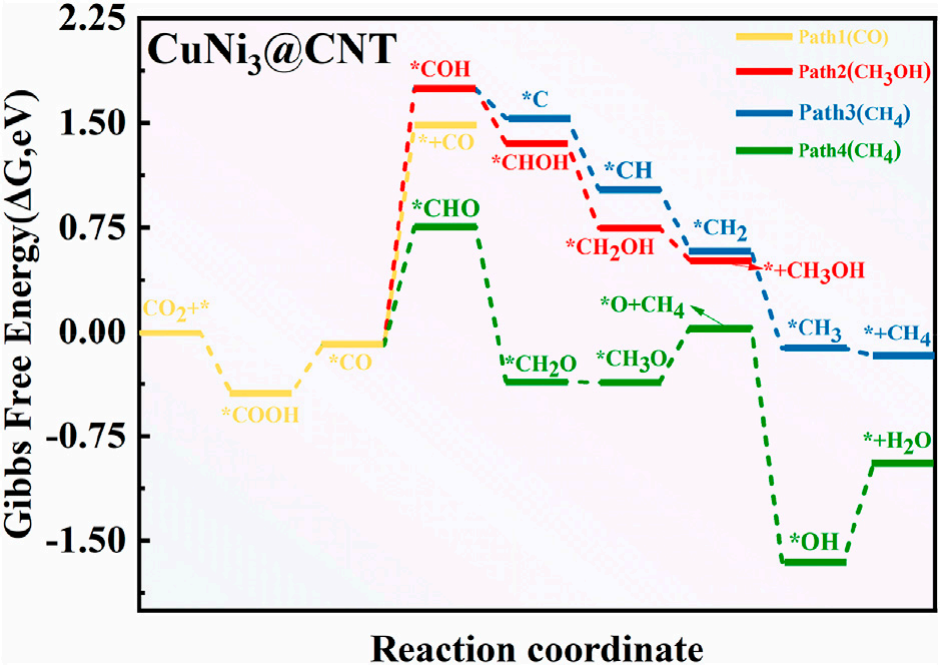
Free energy diagram of CO_2_RR on CuNi_3_@CNTs.

**FIGURE 9:**
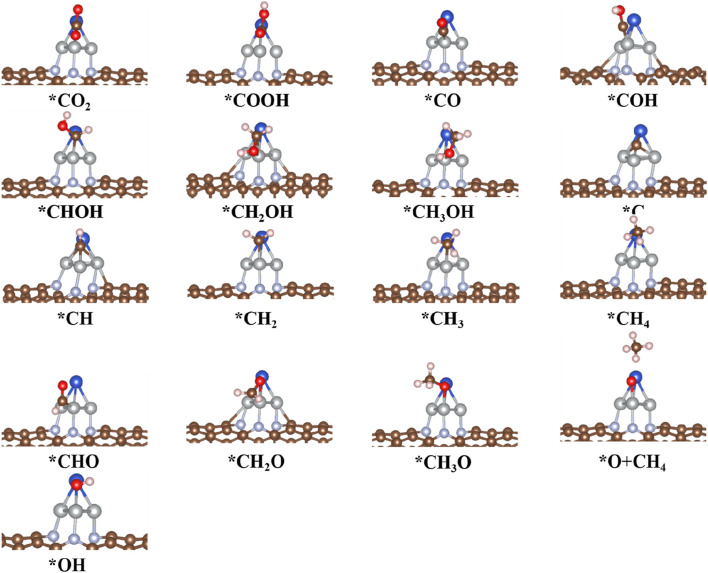
Optimized structure of intermediates for CO_2_RR on the CuNi_3_@CNT surfaces.

To explore the composition effect on the mechanism of CO_2_RR, the synthesis paths of CO_2_ into CH_4_, CO, and CH_3_OH products were determined on CuNi_3_@CNTs. The free energy and geometric structure diagrams are shown in Figures 8, 9, respectively. The *COOH intermediate is obtained in the first step of hydrogenation of the adsorbed *CO_2_ molecule, with a downhill energy of −0.44 eV on the CuNi_3_@CNTs catalyst, which differs from that on Cu@CNTs and Cu_4_@CNTs. According to the aforementioned charge transfer analysis, the adsorbed *CO_2_ molecule obtains more electrons from CuNi_3_@CNTs compared to Cu@CNTs and Cu_4_@CNTs, and the electrons of Ni-3*d* orbitals are partly transferred to the π anti-bonding orbitals of CO_2_. These strong electron transfers lead to the spontaneous formation of a *COOH intermediate. The production of *CO occurs through *COOH hydrogenation to *CO and H_2_O molecules. However, *CO desorption requires a large desorption energy of 1.57 eV (Path 1). Thus, *CO is further hydrogenated to *COH (Δ*G =* 1.83 eV) or *CHO (Δ*G =* 0.84 eV). In the subsequent hydrogenation steps, *CHO is hydrogenated in two steps to form *CH_2_O and *CH_3_O in Path 4. Then, the (H^+^/e^−^) pair attacks the C atom in *CH_3_O, resulting in CH_4_ formation. After release of the CH_4_ molecule, one *O atom remains above the bridge site of the Cu–Ni atoms (Figure 9) and is subsequently further hydrogenated into H_2_O molecules and released. The reactions are more likely to follow Path 2 to form CH_3_OH than Path 3 to form CH_4_ based on a comparison of the Δ*G* values of producing possible species (*CHOH, *CH_2_OH; *C, *CH_2_, and *CH_3_). As discussed previously, CO_2_RR is more likely to follow Path 4 to form CH_4_ on CuNi_3_@CNTs, with an overpotential value of 0.60 V and *COH formation being the PDS. For the Cu–Ni alloy catalysts, the major products of CO_2_RR change from ethylene (C_2_H_4_) and C_2_H_5_OH to CH_4_ with increasing Ni content (Park et al., 2021). These selectivity challenges are attributed to the increased adsorption energies of *CO on the Ni surface based on the results of pressure-dependent experiments and DFT calculations. We observed similar findings in our calculations. The *E*_ads_ values of *CO on the CuNi_3_@CNTs are much higher than those on the Cu@CNTs and Cu_4_@CNTs. Thus, the introduction of Ni atoms in the CuNi_3_@CNTs changes the selectivity of CO_2_RR to produce CH_4_ compared to that on the Cu_4_@CNTs, which produce CO.

### 3.6 Competitive relationship of CO_2_RR vs*.* hydrogen evolution reaction (HER)

**FIGURE 10:**
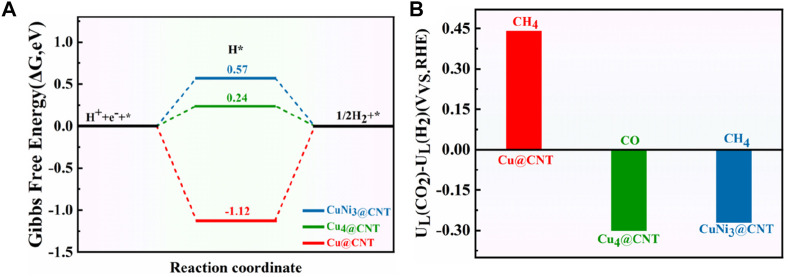
**(A)** Free energy diagram of the HER on Cu@CNTs, Cu_4_@CNTs, and CuNi_3_@CNTs. **(B)** Differences in limiting potentials for CO_2_RR and HER on Cu@CNTs, Cu_4_@CNTs, and CuNi_3_@CNTs.

The hydrogen evolution reaction (HER) is usually considered a competitive reaction during CO_2_RR. Therefore, the selectivity and activity of CO_2_RR are tightly related to HER performance (Xue et al., 2020; Zhao and Liu, 2020). Figure 10A shows the free energies of HER for Cu@CNTs, Cu_4_@CNTs, and CuNi_3_@CNs. HER occurs on three catalysts with Δ*G* values of −1.12, 0.24, and 0.57 eV, respectively. The difference in limiting potentials for CO_2_RR and HER on Cu@CNTs, Cu_4_@CNTs, and CuNi_3_@CNTs were also calculated to further understand the selectivity of the catalyst for CH_4_ or CO formation. The optimized configurations of *H adsorption are shown in Supplementary Figure S3. The higher the *U*_L_ (CO_2_)–*U*_L_ (H_2_) difference, the higher is the selectivity for CO_2_RR over HER (Shen et al., 2021; Wei et al., 2021). As shown in Figure 10B, the selectivity toward CO_2_RR decreases in the order of Cu@CNTs (CH_4_ product) > CuNi_3_@CNTs (CH_4_ product) > Cu_4_@CNTs (CO product). The Cu@CNTs have only one adsorption site for the intermediates, while the CuNi_3_@CNTs and Cu_4_@CNTs have multiple adsorption sites (top, edge, and hollow sites). Thus, smaller-sized Cu@CNTs show the highest CH_4_ selectivity among the three catalyst types. However, the large size of the bulk Ni (110) facet shows the highest selectivity for CH_4_ compared to Ni@CNTs and Ni_4_@CNTs among the Ni-based catalysts (Cao, 2020b). In addition, the selectivity of CH_4_ on the CuNi_3_@CNTs is higher than that for CO on the Cu_4_@ CNTs. Therefore, the sizes and composition of Cu-based catalysts have a significant role in CO_2_RR selectivity.

## 4 Conclusion

In summary, the size and composition effect for the activity and selectivity of CO_2_RR were explored using DFT. Three types of Cu-based catalysts (Cu@CNTs, Cu_4_@CNTs, and CuNi_3_@CNTs) were designed to achieve highly efficient CO_2_ reduction. The CuNi_3_@CNTs show the largest CO_2_ adsorption energy (−0.82 eV) due to CO_2_ molecule adsorption on the edge site between the Ni and Cu atoms. This promotes higher charge transfer to CO_2_ molecules and weakens the C=O double bond of CO_2_. With a size increase from single-atom Cu to the Cu_4_ cluster, the CO_2_RR product changes from CH_4_ to CO. More importantly, Cu_4_@CNTs show an extremely low overpotential of 0.02 V for CO formation, while Cu@CNTs exhibit the highest selectivity for CH_4_ formation among the three catalysts. When the Ni atoms are introduced in the Cu_4_ cluster, the CO_2_RR selectivity is improved compared to HER. The activity and selectivity of the Cu-based catalysts for the CO_2_RR depended strongly on the size and composition. Our results provide new insight into understanding the size and alloy effect of CO_2_RR on Cu-based catalysts.

## Data Availability

The original contributions presented in the study are included in the article/Supplementary Material; further inquiries can be directed to the corresponding authors.
