# Supplementary material for: Structure–activity relationship of Cu-based catalysts for the highly efficient CO2 electrochemical reduction reaction
Source: Front Chem. 2023 Feb 9;11:1141453. doi: 10.3389/fchem.2023.1141453 (PMC9947715; doi:10.3389/fchem.2023.1141453)
Supplement: Supplementary file 1 [file DataSheet1.docx]

**Structure–activity relationship of Cu-based catalysts for highly efficient CO_2_ Electrochemical Reduction Reaction**

*Runzhi An^#, 1^, Xuanqi Chen^#, 1^, Qi Fang^1^, Yuxiao Meng^1,2^, Xi Li ^*,1^, Yongyong Cao^*,1^*

*^1^ College of Biological, Chemical Science and Engineering Jiaxing University, Jiaxing, Zhejiang 314001, PR China*

*^2^ College of Chemical Engineering, State Key Laboratory Breeding Base of Green-Chemical Synthesis Technology, Zhejiang University of Technology, Hangzhou 310032, PR China*

**Computational Results**


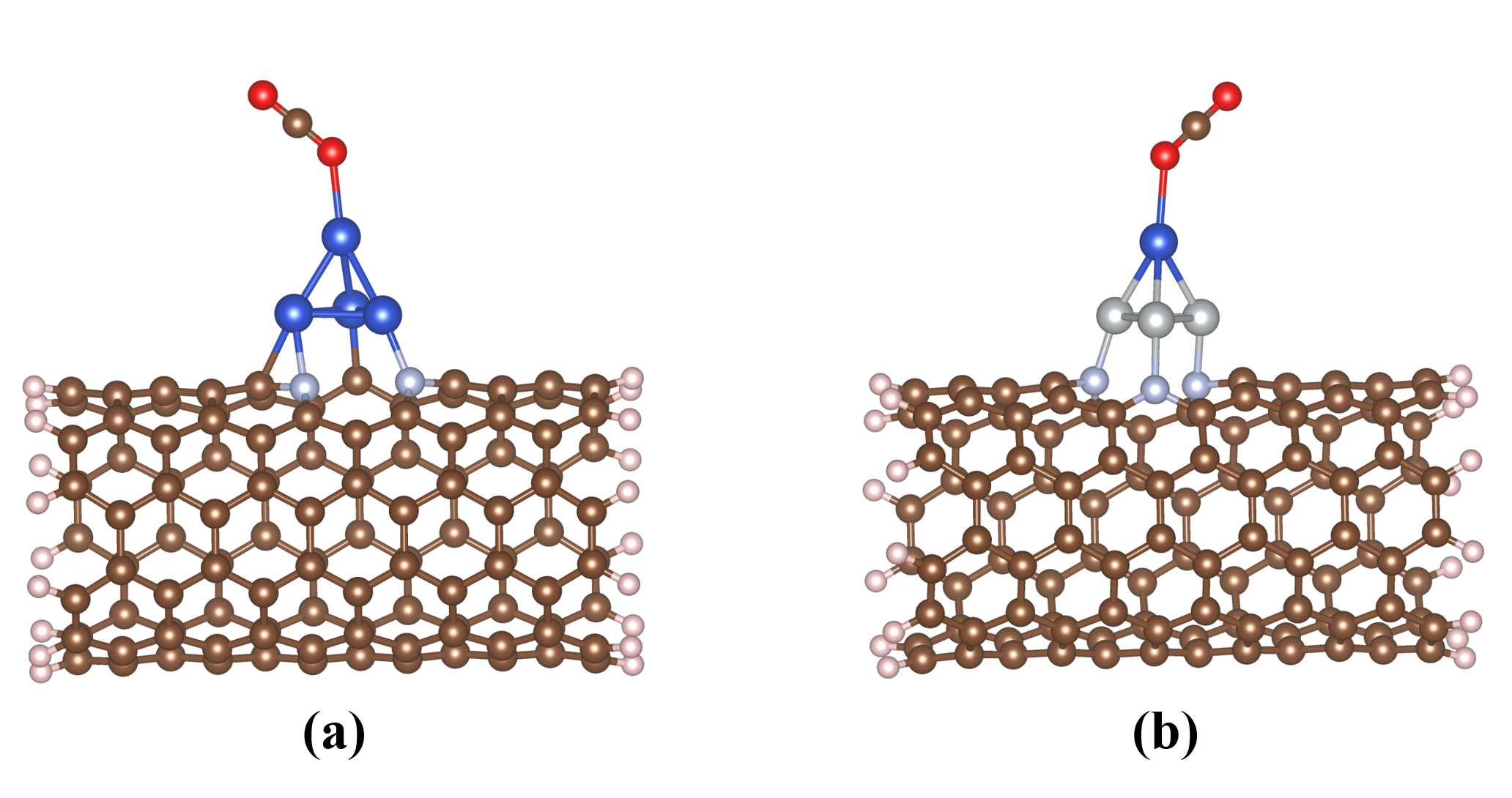


**FIGURE S1** the optimized CO_2_ molecule adsorption on the top site of (a) Cu_4_@CNTs and (b) CuNi_3_@CNTs, respectively.


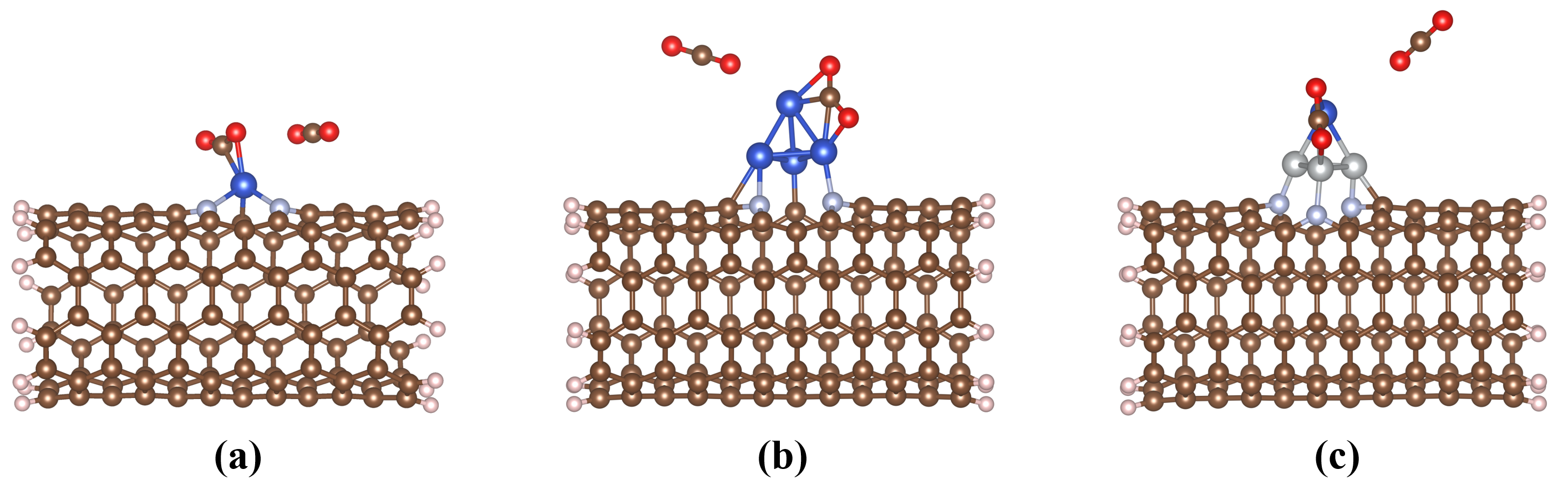


**FIGURE S2** the optimized two CO_2_ molecule co-adsorption on the (a) Cu@CNTs, (b) Cu_4_@CNTs and (c) CuNi_3_@CNTs, respectively.


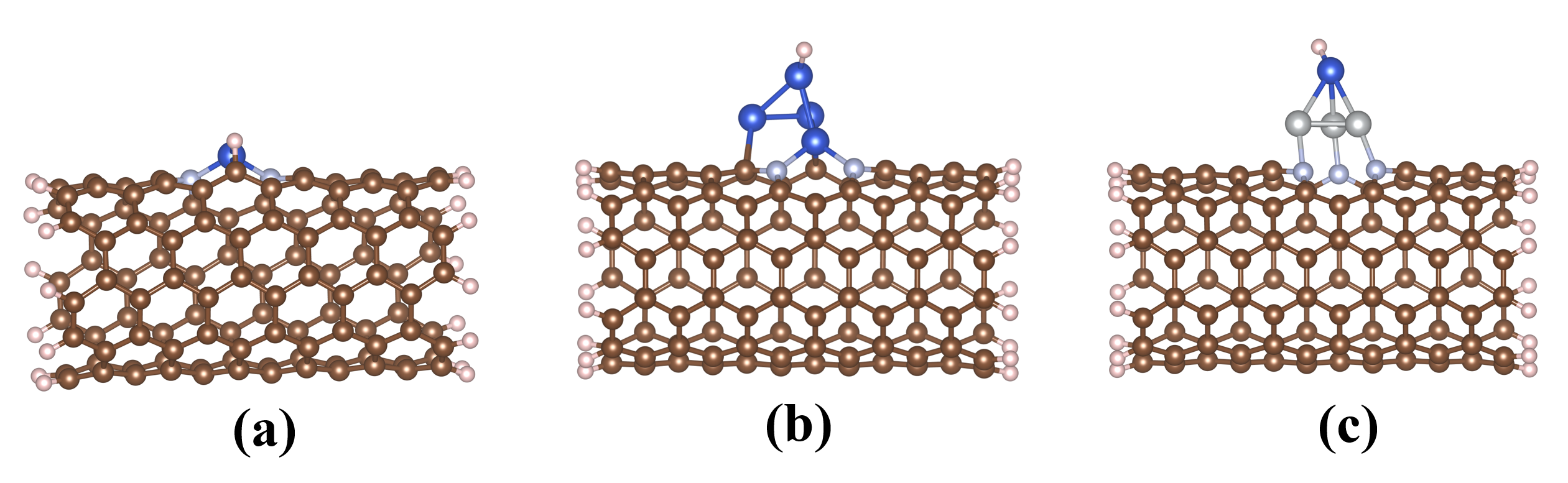


**FIGURE S3** The optimized structure of *H adsorption on (a) Cu@CNTs, (b) Cu_4_@CNTs and (c) CuNi_3_@CNTs surface.
