# Supplementary material for: Structure–activity relationship of Cu-based catalysts for the highly efficient CO2 electrochemical reduction reaction
Source: Front Chem. 2023 Feb 9;11:1141453. doi: 10.3389/fchem.2023.1141453 (PMC9947715; doi:10.3389/fchem.2023.1141453)
Supplement: Supplementary file 2 [file Image1.pdf]

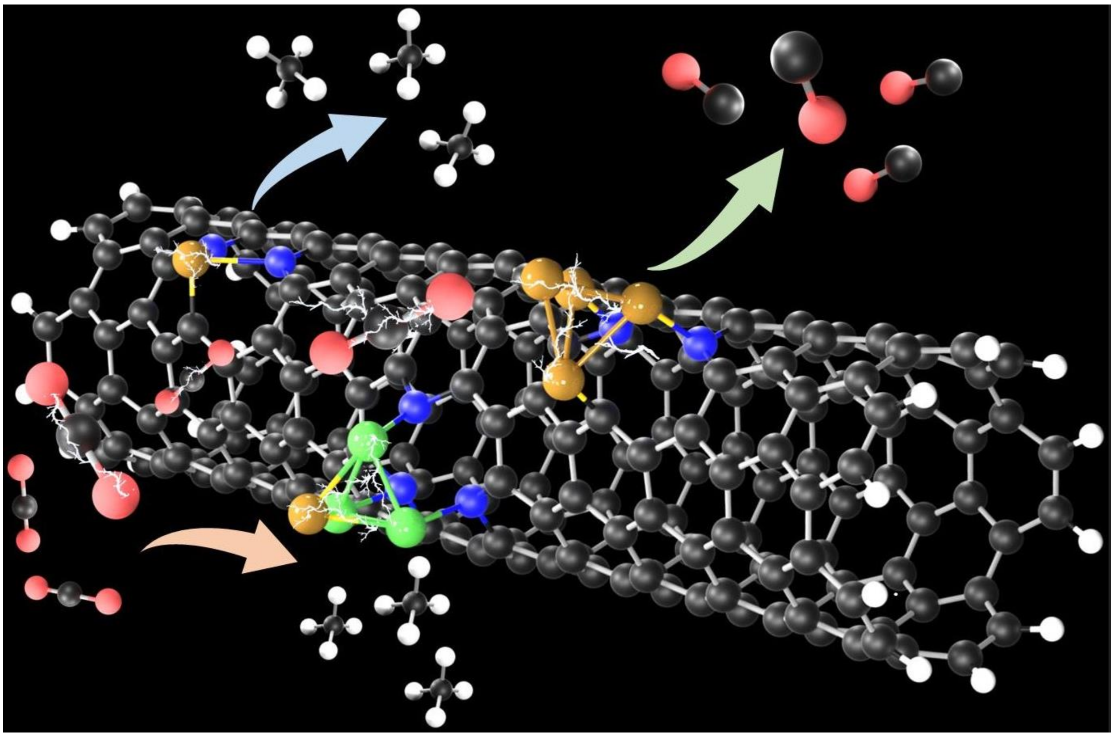

The sizes and compositions of Cu-based catalysts affected the activity and selectivity for CO_2RR by density functional theory calculations.
